# Step-by-step and orderly lowering of the height of inferior vena cava tumor thrombus is the key to robot-assisted thrombectomy for Mayo III/IV tumor thrombus

**DOI:** 10.1186/s12885-022-09235-7

**Published:** 2022-02-07

**Authors:** Guo-Dong Zhao, Xiu-Ping Zhang, Ming-Gen Hu, Qing-Bao Huang, Shuai Xu, Bao-Jun Wang, Xin Ma, Xu Zhang, Wen-Bo Zou, Xuan Zhang, Zhi-Ming Zhao, Xiang-Long Tan, Sai Chou, Gang Wang, Rong Liu

**Affiliations:** 1 Faculty of Hepato-Biliary-Pancreatic Surgery, Chinese People’s Liberation Army (PLA) General Hospital, Institute of Hepatobiliary Surgery of Chinese PLA, 28 Fuxing Road, Beijing, 100853 China; 2 Faculty of Urology Surgery, Chinese People’s Liberation Army (PLA) General Hospital, Beijing, China; 3 School of Medicine, Nankai University, Tianjin, China

**Keywords:** Step-by-step and orderly, Robot-assisted thrombectomy, Mayo III/IV level, Inferior vena cava tumor thrombus

## Abstract

**Background:**

The surgical management of Mayo III/IV tumor thrombi is difficult and risky, and robotic surgery is even more difficult. The purpose of this study was to introduce the step-by-step and orderly lowering of the height of inferior vena cava tumor thrombus, which was the core technique of robot operation for Mayo III/IV tumor thrombus.

**Method:**

A total of 18 patients were included in this study. The average tumor thrombus height was 2.4 cm above the level of the second porta hepatis (SPH), and 9 patients were prepared for cardiopulmonary bypass (CPB) before surgery. During the operation, the height of the tumor thrombus was lowered orderly for 2-3 times, and the blood flow blocking method was changed sequentially. The CPB was required when tumor thrombus in the atrium; After the height of the thrombus was lowered to the atrium entrance, CPB was stopped and the blood flow was blocked in the upper- and retro-hepatic inferior vena cava (IVC); After the tumor thrombus continued to descend to the lower part of the SPH, liver blood flow could be restored, and then, the blood flow was simply blocked in the retro-hepatic IVC to complete the removal of the thrombus and the repair or resection of the IVC. Finally, the diseased kidney and renal vein were removed.

**Results:**

All operations were successfully completed, and 2 cases were transferred to laparotomy. Seven cases received CPB, while the other 11 did not. 15 patients underwent two times of the lowering of the tumor thrombus, 2 patients underwent one time and 1 patient underwent three times. The mean liver/IVC dissociation and vascular suspension time was 22.0 min. All patients had less than Clavien-Dindo grade III complications, no serious complications occurred during operation, and no patient died within 90 days.

**Conclusions:**

The step-by-step and orderly decline of tumor thrombus height is the key to the success of robot Mayo III / IV tumor thrombus surgery. This method can shorten FPH and CPB time and improve the success rate of surgery.

## Background

The treatment of inferior vena cava tumor thrombus (IVCTT) is challenging and rarely involves minimally invasive surgery. In our center, Zhang’s team organized multidisciplinary cooperation to conduct in-depth clinical and basic scientific research on IVCTT of the renal tumor and accumulated a wealth of experience. Zhang’s team systematically introduced the surgical treatment strategy of robot-assisted thrombectomy for IVCTT by publishing a series of papers [1–3], and they have received attention and recognition from their peers. For Mayo level I and II IVCTT, minimally invasive surgical treatment is relatively easy [4–6], and the operating procedures are also relatively uniform [7, 8]. For Mayo level III/IV IVCTT, high IVCTT, including the level of the hepatic vein, suprahepatic/subdiaphragmatic and supradiaphragmatic/subatrial IVCTT, due to the need for multispecialty cooperation, the establishment of abundant collateral circulation of the inferior vena cava and the significant widening of the inferior vena cava, minimally invasive operation is extremely difficult [9, 10]. Traditional laparoscopy is very hard to perform. Although robotic surgery has been proven to be technical feasible, there is a shortage of clinical reports [11, 12]. Moreover, most of the articles focus on level III tumor thrombus, and only our center has reported on level IV tumor thrombus [2, 13, 14]. Most studies lack a detailed description of the operation technology and repeatability of the methods, especially in the high inferior vena cava dissociation, suspension, occlusion and thrombectomy. Additionally, given the progress of the anatomical understanding of the high inferior vena cava and the improvement in surgical strategies, timely adjustment to the traditional Mayo grade is needed to better guide increasingly optimized clinical surgery [15].

The author is a professional hepatobiliary surgeon and an important member of the multidisciplinary team who has accumulated abundant experience in robotic surgery for the vast majority of renal tumors with IVCTT. From the view of hepatobiliary surgeons, this study briefly discusses the step-by-step and orderly lowering of the height of IVCTT as the key to robot-assisted thrombectomy for Mayo III/IV tumor thrombus. Additionally, according to the differences in various surgical strategies, proposed modifications to the Mayo grade, which also provided a reference for the surgical treatment of retroperitoneal tumor, liver cancer and other tumors with IVCTT [16, 17], are offered.

## Methods

### Patient selection and study design

From January 2017 to now, our team has participated in the completion of the Mayo III/IV IVCTT 18 cases in the Chinese PLA General Hospital in Beijing, China. According to the patients’ preoperative examinations, we divided them into two groups, including 11 cases in the noncombined-cardiopulmonary bypass (CPB) group and 7 cases in the CPB group(9 cases may be performed CPB in preoperative planning, but 2 not done finally because of height of tumor thrombus above the level of the second hilar less than 3 cm). All these surgical operations were performed by the same multidisciplinary team. Data on their demographic characteristics and perioperative outcomes were collected and analyzed. Follow-ups were conducted using laboratory tests and abdominal ultrasonography. Patients who underwent robot-assisted thrombectomy were followed-up once every 2-3 months until death. This study was approved by the Institutional Review Committee of the Chinese PLA General Hospital. Each patient signed an informed consent form as an agreement to accept the operation, and their preoperative and postoperative data were used in this study.

### Operative procedures

During the operation, the height of tumor thrombus was decreased orderly for 2-3 times, and the blood flow blocking method was changed in order to complete the operation. If the tumor thrombus is in the atrium, cardiopulmonary bypass is first needed; After the height of tumor thrombus decreased to the atrial entrance, cardiopulmonary bypass was stopped and the blood flow in suprahepatic and retrohepatic inferior cavities was blocked; After the height of the tumor thrombus continues to fall below the second hepatic portal, the liver blood flow can be restored, changed to simple retrohepatic inferior vena cava blood flow occlusion, complete the removal of the tumor thrombus and the repair or resection of the inferior vena cava, and finally remove the diseased kidney and renal vein.

### Surgical procedures for III and low IV tumor thrombi without combining CPB intraoperatively (left renal tumor with IVCTT is used as an example)

**Fig. 1:**
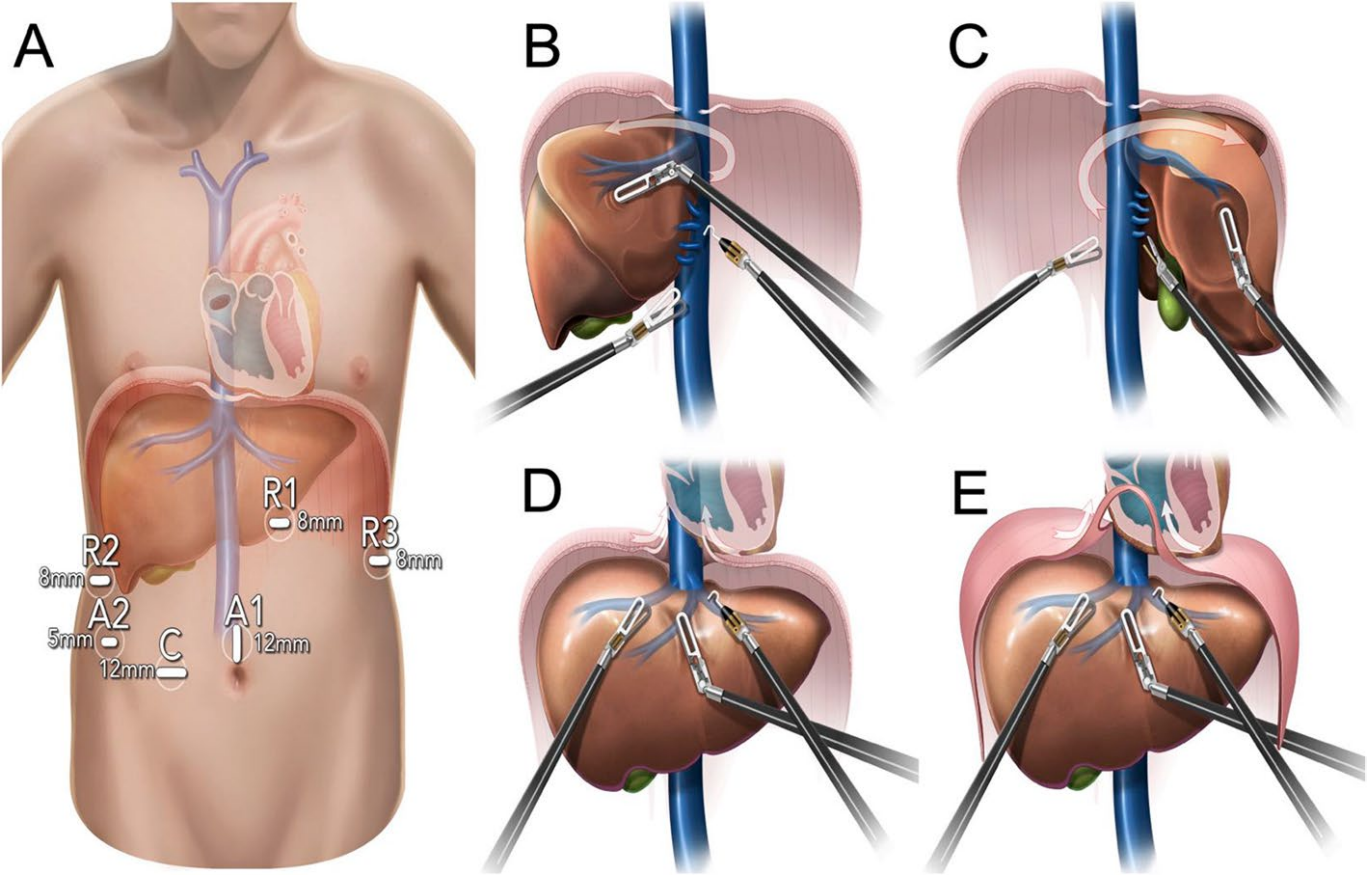
**A** The conventional port placements of robotic liver surgery; High segment of liver of the IVC were dissociated in “left-right-superior” order. **B** The left short hepatic vessel was severed successively to the level of the second porta hepatis (SPH) (left). **C** After that, the right triangular ligament, coronal ligament and hepatorenal ligament were incised in turn (right). **D** Opening the fibrous connection between the diaphragm and the inferior vena cava, entered the mediastinum from the foot side. **E** if necessary, the ventral diaphragm and pericardium of the second hepatic hilum were opened longitudinally to expose the IVC, atrium and ventricle of the whole liver atrium, then dissociated the suprahepatic and subatrial segments of the IVC

First, the operation was performed by the hepatobiliary surgeon. The patient was in a split-legged supine position in a dorsal elevated position. The right hypochondrium was slightly elevated, and the conventional port placements of robotic liver surgery were used (Fig. 1A) in the operation. High segment of liver of the IVC were dissociated in “left-right-superior” order. First, the ligamentum teres hepatis, falciform ligament, and left deltoid ligament were severed. Then, we opened the peritoneum adjacent to the inferior vena cava from the foot side to the cephalic side. The left short hepatic vessel was severed successively to the level of the second porta hepatis (SPH) (left, Fig. 1B). After that, the right triangular ligament, coronal ligament and hepatorenal ligament were incised in turn. We used the traction arm to gradually flip the right liver to the left, severed the right short hepatic vessels successively from the foot side to the cephalic side, and dissociated the right side of the inferior vena cava at the level of the SPH (right, Fig. 1C). Then, we opened the fibrous connection between the diaphragm and the inferior vena cava, entered the mediastinum from the foot side (Fig. 1D), if necessary, the ventral diaphragm and pericardium of the second hepatic hilum were opened longitudinally to expose the IVC, atrium and ventricle of the whole liver atrium, then dissociated the suprahepatic and subatrial segments of the IVC (Fig. 1E). Sling operations were performed above the tumor thrombus and below the SPH and first porta hepatis (FPH). Additionally, the location of the tumor thrombus and the sling was repeatedly confirmed by laparoscopic ultrasound before the sling operation.

After the high inferior vena cava was full exposure and suspension, the urologist continued to operate, and the patient’s position was changed from the supine to the left lateral decubitus position and ports were rearranged. After disconnecting the left renal vein (LRV), IVC, right renal artery (RRA), right renal vein (RRV) and FPH below the tumor thrombus (to occlude the FPH) and the IVC above the tumor thrombus was successively occluded, we incised the lateral IVC and lowered the height of the tumor thrombus to below the SPH (the first drop in the position of the tumor thrombus, 1st Drop, release the FPH, and occlude retrohepatic inferior vena cava). Amputated, sutured or implemented suspension was used to block the IVC below the SPH, release the FPH and unlock the sling above the SPH in turn to restore liver blood flow and gastrointestinal blood reflux (release the FPH), with continued thrombectomy or en bloc resection to the retrohepatic inferior vena cava and root of the left renal vein (the second drop in the position of the tumor thrombus, 2^nd^ Drop). The position was further adjusted to the right lateral decubitus position, and finally, we removed the diseased kidney.

The surgical strategy of the right renal tumor with IVCTT is slightly different from that of the left in the intraoperative sequence and mode of vascular occlusion; it has less postural adjustment. Similarly, the liver supine position and conventional robotic port placements were used, and liver dissociation and vascular suspension were performed first. We further adjusted the position to the left lateral decubitus position, blocked the LRV and IVC below the tumor thrombus, as well as the FPH and IVC above the tumor thrombus, in turn, without blocking the LRA. Then, thrombectomy and resection of the inferior vena cava could be performed. Finally, we removed the diseased kidney.

### Surgical procedures for high-IV tumor thrombus combined with CPB during operation (all subjects included in this study were patients with the right renal tumor)

First, the operation was performed by the hepatobiliary surgeon, and the same steps were taken as before. Each patient was in the liver position, and conventional port placements for robotic liver surgery were used in the operation. We dissociated the liver and suspended blood vessels in left-right-superior order. The same sling operations were performed above the SPH and below the SPH and FPH. Moreover, intraoperative ultrasound further defined the height of the tumor thrombus. After confirming that the IVC above the tumor thrombus could not be effectively and safely transabdominally blocked, we decided to establish CPB.

The cardiovascular surgeon continued to perform surgery and prepared for the establishment of CPB. We intubated the internal jugular vein and the right femoral artery and vein, dissociated and suspended the SVC by a right fifth intercostal small incision, dissociated IVC below the SPH from the thoracic cavity, and properly adjusted the position of the sling retained with the help of a hepatobiliary surgeon.

The patients’ position was changed from the supine to the left lateral decubitus position, RRV and IVC below the tumor thrombus were dissociated. Urologists cooperated with cardiovascular surgeons to occlude the SVC, FPH, LRV and IVC below the tumor thrombus successively (establish the CPB and occlude the FPH). A cardiovascular surgeon performed atrial and (or) IVC thrombectomy, removed as cleanly as possible the tumor thrombus inside the atrium and above the SPH and minimized the height of the tumor thrombus. Urologists transabdominally dissected the retrohepatic IVC, further lowered the height of the tumor thrombus to below the SPH using an up-down combination (the first drop in the position of the tumor thrombus, 1^st^ Drop). Sutured or amputated IVC positioned below the SPH and the blocked band of FPH and IVC above the SPH were released, restoring liver blood flow and gastrointestinal blood reflux (stop the CPB and release the FPH, and occlude retrohepatic inferior vena cava). We used plasma substitutes or vasoactive drugs promptly to correct coagulation function. We continued to conduct en bloc resection of the retrohepatic inferior vena cava (the second drop in the position of the tumor thrombus). Finally, we removed the diseased kidney and put the venous tumor thrombus and the diseased kidney in the specimen bag together, removing it from the upward expansion of the incision at the belly button.

When some of the high tumor thrombi could not fall below the SPH at one time, we used the strategy of lowering the tumor thrombus three times. First, under cardiopulmonary bypass (establish the CPB and occlude the FPH), the cardiovascular surgeon removed the tumor thrombus above the sling of the SPH and lowered the tumor thrombus to below the sling of the SPH (the first drop in the position of the tumor thrombus), occluding the IVC above the SPH (stop the CPB and continue to occlude the FPH, and occlude retrohepatic and superior and inferior hepatic vena cava). After that, we further lowered the height of the tumor thrombus to below the SPH (the second drop in the position of the tumor thrombus to stop to occlude the FPH, and occlude retrohepatic and inferior hepatic vena cava), sutured or amputated the IVC below the SPH, released the FPH and IVC above the SPH in turn (release the FPH). When the patients’ circulation was well-established, the involved inferior vena cava could be removed directly. If collateral circulation was not established, thrombectomy for the IVC and vascular repair should be conducted.

## Results

### Patient characteristics

According to the patients’ preoperative examinations, we divided them into two groups, including 11 cases in the noncombined-CPB group and 7 cases in the CPB group. 10 and 8 patient were diagnosed as level III (Beyond the SPH ≤3cm) and IV (Beyond the SPH >3cm), respectively. The patient characteristics are displayed in Table 1.

**Table 1:** General information of patients with Mayo grade III or higher tumor thrombi who underwent high inferior vena cava cancer thrombectomy by robot ^a^

Characteristic	No CPB	CPB	Total
**Cases, n (%)**	11 (100.0)	7 (100.0)	18 (100.0)
**Sex, n (%)**			
Male	6 (54.5)	5 (71.4)	11 (61.1)
Female	5 (45.5)	2 (28.6)	7 (38.9)
**Age (years), mean (SD)**	55.1 (12.3)	56.5 (12.2)	55.7 (11.9)
**BMI (kg/m**^**2**^**), mean (SD)**	24.0 (2.9)	23.0 (2.4)	23.6 (2.7)
**Affected kidney, n (%)**			
Left	5 (45.5)	2 (28.6)	7 (38.9)
Right	6 (54.5)	5 (71.4)	11 (61.1)
**Preoperative neoadjuvant therapy, n (%)**	2 (18.2)	1 (14.3)	3 (16.7)
**Preoperative angiography, n (%)**	3 (27.3)	2 (28.6)	5 (27.8)
**Conventional Mayo stage**			
III			10
IV			8
**Modified Mayo stage** ^**b**^**, n (%)**			
Beyond the SPH ≤3cm	10 (90.9)	0 (0.0)	10 (55.6)
Beyond the SPH >3cm	1 (9.1)	7 (100.0)	8 (44.4)
**TNM stage, n (%)**			
1	0 (0.0)	0 (0.0)	0 (0.0)
2	0 (0.0)	0 (0.0)	0 (0.0)
3	10 (90.9)	7 (100.0)	17 (94.4)
4	1 (9.1)	0 (0.0)	1 (5.6)
**Tumor diameter (cm), mean (SD)**	8.9 (2.9)	7.8 (2.6)	8.5 (2.8)
**Length of cancer thrombus in IVC (cm), mean (SD)**	9.5 (1.8)	7.1 (3.9)	8.4 (3.1)
**Diameter of cancer thrombus in IVC (mm), mean (SD)**	30.2 (11.0)	27.7 (12.2)	29.2 (11.2)
**Height above the SPH of cancer thrombus** ^**c**^ **(cm), mean (SD)**	1.3 (1.2)	4.1 (0.8)	2.4 (1.7)
**Preoperative angioembolization, n (%)**	3 (27.3)	2 (28.6)	5 (27.8)

*Abbreviations*: *CPB* Cardiopulmonary bypass, *BMI* Body Mass Index, *IVC* inferior vena cava, *SD* standard deviation, *SPH* second porta hepatis

*Note:*

^a^ The height of inferior vena cava cancer thrombus higher than the lower margin of the SPH is called high inferior vena cava cancer thrombus

^b^ Refer to the Modified Mayo clinical classification (Fig. 3)

^c^ When the tumor enters the right atrium, here we calculate the height of the lower margin of the SPH to the entrance of the right atrium

### Surgical outcomes and prognosis

**Fig. 2:**
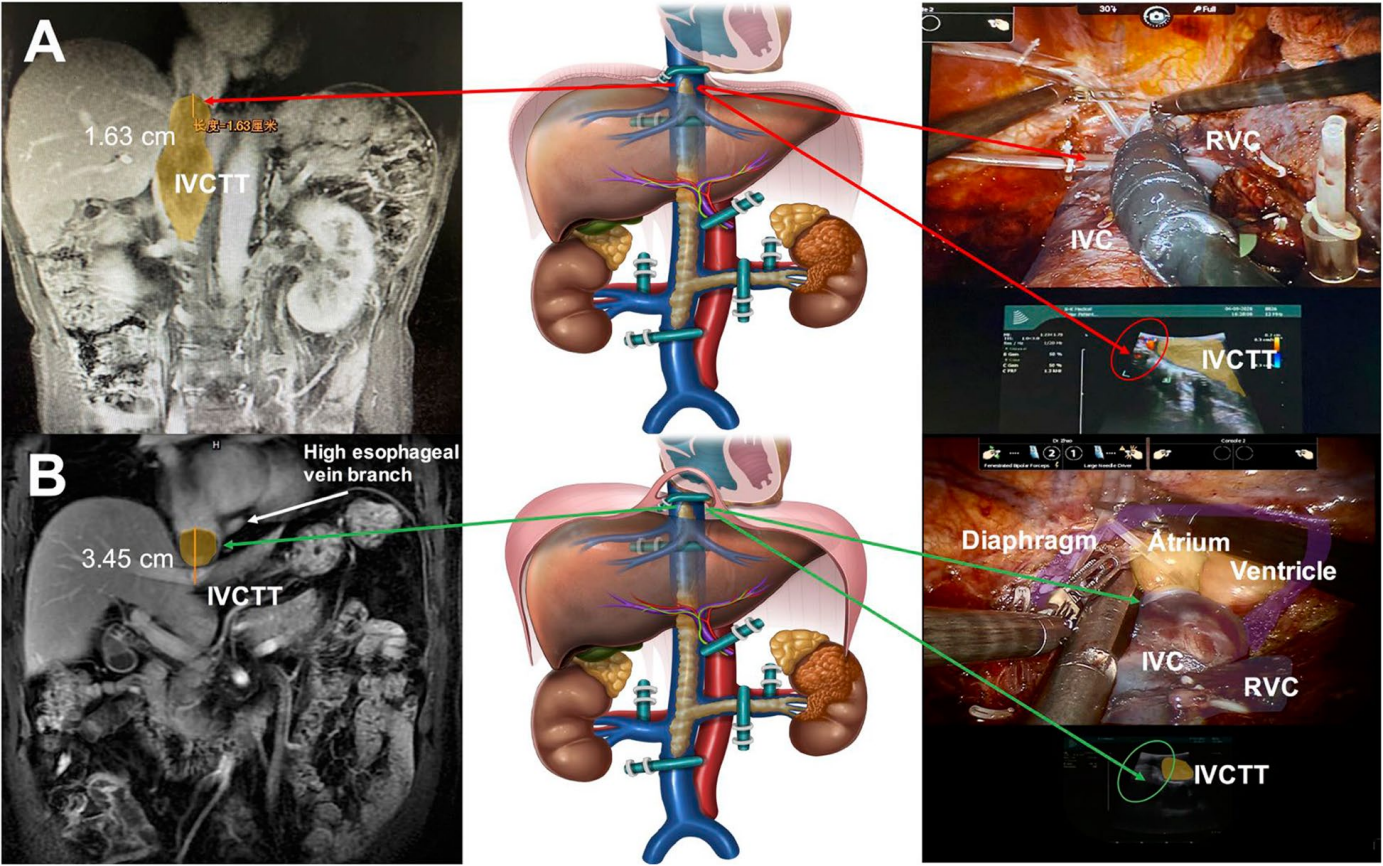
Two examples of patients with different level IVCTT were shown (**A** 1.63cm beyond diaphragmatic level; **B** 3.45cm beyond diaphragmatic level)

**Fig. 3:**
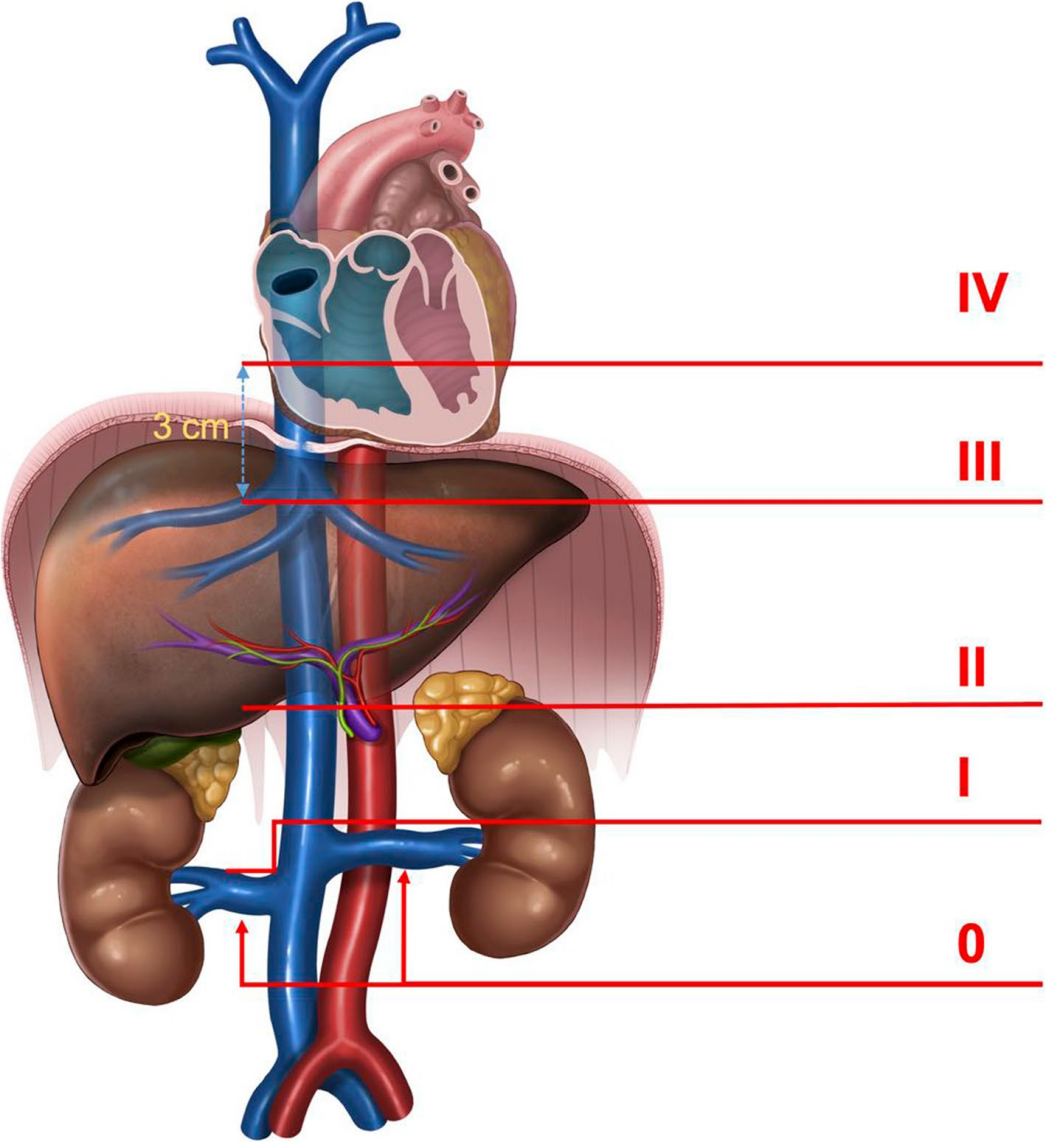
Suggestions on revision of Mayo classification

A total of 18 patients underwent robot-assisted thrombectomy for Mayo III/IV tumor thrombus. Eleven patients did not undergo CPB, and all patients received sling suspension above the SPH. Two examples of patients with different level IVCTT were shown Fig. 2. Surgical outcomes and prognosis are shown in Table 2. When the tumor thrombus was more than 3 cm above the level of the SPH but did not enter the atrium, some of them were also unnecessary to establish CPB (Fig. 3 were suggestions on revision of Mayo classification).

**Table 2:** Surgical outcomes and prognosis of patients undergoing robotic thrombectomy for high inferior vena cava cancer thrombus ^a^

Characteristic	No CPB	CPB	Total
**Surgical outcomes**			
**Height of the cancer thrombus, n (%)**			
Limited in IVC	11 (100.0)	0 (0.0)	11 (61.1)
Into the right atrium	0 (0.0)	7 (100.0)	7 (38.9)
**Transfer to laparotomy, n (%)**			
Yes	2 (18.2)	0 (0.0)	2 (11.1)
No	9 (81.8)	7 (100.0)	16 (88.9)
**Times of cancer thrombus drops, n (%)**			
1	2 (18.2)	0 (0.0)	2 (11.1)
2	9 (81.8)	6 (85.7)	15 (83.3)
3	0 (0.0)	1 (14.3)	1 (5.6)
**Liver/IVC dissociation and vascular suspension time (min), median (IQR)**	20 (14-26)	29 (20-33)	22 (14-32)
**Blood loss during Liver /IVC dissociation and blood vessel suspension (ml), median (IQR)**	400 (200-750)	600 (250-1200)	530 (230-1000)
**Operative time (min), median (IQR)**	355.0 (305.0-465.0)	540.0 (525.0-593.3)	465.0 (338.0-540.0)
**Intraoperative blood loss (ml), median (IQR)**	1000.0 (950.0-2500.0)	2750.0 (1375.0-5250.0)	1500.0 (1000.0-3500.0)
**Intraoperative transfusion (ml), median (IQR)**	1200.0 (600.0-1230.0)	3320.0 (1300.0-4215.0)	1200.0 (990.0-3240.0)
**Postoperative recovery and prognosis**			
**Postoperative ICU transition days (d), mean (IQR)**	2 (1-4)	3 (1-6)	3 (2-5)
**Postoperative drainage time (d), mean (IQR)**	4 (3-7)	5 (4-7)	5 (3-7)
**Postoperative hospital stay (d), mean (IQR)**	8.0 (6.0-11.5)	11.5 (9.3-14.4)	10.0 (7.0-13.0)
**Perioperative complications** ^**d**^**, n (%)**			
≤IIIa	11 (100.0)	7 (100.0)	18 (100.0)
≥IIIb	0 (0.0)	0 (0.0)	0 (0.0)
**90-day mortality, (%)**	0	0	0
**Follow-up (12 Month), n (%)**			
Alive	5 (45.5)	3 (42.9)	8 (44.4)
Dead	1 (9.0)	1 (14.3)	2 (11.2)
Loss to follow-up	5 (45.5)	3 (42.9)	8 (44.4)
**Pathologic types, n (%)**			
ccRCC	9 (81.8)	6 (85.7)	15 (83.3)
UC	1 (9.1)	0 (0.0)	1 (5.6)
PRCC	1 (9.1)	1 (14.3)	2 (11.1)
**Fuhrman stage, n (%)**			
I	0 (0.0)	0 (0.0)	0 (0.0)
II	5 (45.4)	1 (14.3)	6 (33.4)
III	4 (36.4)	4 (57.1)	8 (44.4)
IV	2 (18.2)	2 (28.6)	4 (22.2)

*Abbreviations*: *CPB* Cardiopulmonary bypass, *IVC* inferior vena cava, *IQR* interquartile range, *Hb* hemoglobin, *ALB* albumin, *AST* Aspartate Aminotransferase, *Cr* Creatinine, *BUN* Blood Urea Nitrogen, *ccRCC* clear cell renal cell carcinoma, *UC* Urothelial Carcinoma, *PRCC* papillary renal cell carcinoma

*Note:*

^a^ The height of inferior vena cava cancer thrombus higher than the lower margin of the SPH is called high inferior vena cava cancer thrombus

^d^ According to the Clavien-Dindo classification of surgical complications

## Discussion

Thrombectomy for high IVCTT, Mayo level III or above tumor thrombus is challenging and risky. The number of centers that can perform the surgery is extremely low, and patients are scarce. Most centers chose traditional open surgery for tumor thrombus and adopted multidisciplinary cooperation and cardiopulmonary bypass during the operation [18, 19]. The multidisciplinary team in which the author works has accumulated a wealth of experience in using robotic surgery for this kind of tumor thrombus. Clinical practice prompts robot-assisted thrombectomy for high IVCTT to be safe and feasible [14, 20–22]. Robotic surgery significantly reduces injury compared to the surgical approach. Meanwhile, robotic surgery makes use of the unique characteristics of lateral visual field of foot and manipulation in a narrow space of endoscopy to improve and simplify the procedure. By lowering the tumor thrombus 2-3 times intraoperatively, it immediately decreases the Mayo level of the tumor thrombus and the difficulty of the operation as well as makes the operation better [1, 4, 23]. This technique has preferable repeatability, and the shortening of the CPB and FPH time can improve the success rate and safety of thrombectomy for high IVCTT. The operation time, intraoperative blood loss, postoperative complications and mortality in all groups were within the acceptable ranges.

The height of IVCTT of the renal tumor is directly related to the difficulty of the operation; the higher the position is, the higher the Mayo level is and the greater the difficulty of the operation is. In contrast, the lower the position of the tumor thrombus is, the lower the difficulty of the operation; hence, the step-by-step and orderly lowering of the height of IVCTT is the key to thrombectomy, and it is also directly relevant to the duration of the CPB and FPH. High IVCTT without establishing CPB can be completed by lowering the height of the tumor thrombus twice, decreasing the level of the tumor thrombus and the difficulty of the operation step by step. First, we lowered the height of the tumor thrombus below the sling beneath the SPH (the first drop in the position of the tumor thrombus) and released the FPH while significantly saving FPH time. We calmly resected the retrohepatic inferior vena cava or removed the tumor thrombus and sutured it (the second drop in the position of the tumor thrombus). Finally, we removed the diseased kidney. For cases combined with CPB, lowering the tumor thrombus twice is the first choice to complete the operation. However, when some retrohepatic and subatrial segments of the IVCTT were longer or the tumor thrombus adhered closely to the wall of the inferior vena cava, we did not lower the tumor thrombus below the SPH at one time. At this time, the operation can be completed by lowering the height of the tumor thrombus 3 times. First, the cardiovascular surgeon removed the thrombus from the cephalic side and cooperated with the urologist, who operated at the ventral side to lower the height of the tumor thrombus below the sling beneath the SPH (the first drop in the position of the tumor thrombus). After occluding the inferior vena cava, CPB was stopped, and the urologist continued to lower the height of the tumor thrombus from the foot side to below the sling beneath the SPH (the first drop in the position of the tumor thrombus). FPH was released, and then we removed the retrohepatic inferior vena cava or the thrombus and sutured it (the first drop in the position of the tumor thrombus). Finally, we resected the diseased kidney.

Some details need be noted intraoperatively. First, the placement of ports is significant in robotic liver surgery. Robotic arm 1 should flip/dissociate over a wide area of the whole liver. Here, the Trocar should have been positioned slightly to the left of the centerline, and its position was fine-tuned by intraoperatively lifting and pulling the abdominal wall. When the Spiegelian lobe is dissociated, the actions of Robotic 1 and 3 arm are interchangeable, with R1 arm as the traction arm and R3 arm as the main operating arms. Second, we need to pay attention to the order of liver dissociation and IVC suspension during operation to improve efficiency and safety. Initially, we dissociated the left liver and the left caudate lobe, completely amputated the left third porta hepatis (TPH), completely exposed the left wall of the retrohepatic inferior vena cava, dissociated the right liver, made use of the “backhand to block the outside” method to flip the right liver by 3 arms and flipped while detaching. After the right liver was completely dissociated, we severed the right short hepatic vein from the foot to the cephalic side and completely dissociated the ventral part of the retrohepatic inferior vena cava. Finally, we separated the fibers between the diaphragm and the suprahepatic IVC to enlarge the vena cava hiatus and dissociated the retrohepatic inferior vena cava from the foot to the entrance of the atrium and below the pericardium (Fig. 2A). If necessary, the pericardium can be opened longitudinally to expose the heart and superior vena cava, then sling operations were performed above the SPH and below the SPH and FPH (Fig. 2B).

When we are operating on both the sides and rear of the retrohepatic inferior vena cava, common venous branches need to be considered, such as the inferior esophageal vein, high esophageal vein branch, phrenic vein, and new collateral vessels [24, 25]; if possible, preoperative vascular 3D reconstruction or angiography can be performed to avoid risk. Among them, the high esophageal vein branch can help to judge the height of tumor thrombus. The tumor thrombus below the vein branch must not enter the atrium, and the probability of needing CPB cooperation is significantly reduced.

CPB and FPH time is one of the factors restricting operation. Intraoperatively, the use of heparin anticoagulation in patients with CPB not only significantly increased the amount of bleeding in the operative area, but also significantly increased the operation time, operative procedure and medical expenses. For Mayo IV tumor thrombus, which is higher than the level of the diaphragm, the traditional method needs the establishment of CPB and requires cooperation between the cardiovascular and the hepatobiliary surgeon. The author's clinical practice suggests that CPB is not needed for some low Mayo IV tumor thrombi. Intraoperatively, the IVC above the SPH and subatrial IVC can be safely transabdominally separated from the foot side to the cephalic side. By lowering the tumor thrombus step by step, the operation can be completed in a short FPH time. Therefore, from the perspective of surgical strategy, the author recommends that the diaphragm should not be classified as ideal anatomical marker to guide grading. Second, strictly speaking, the attachment point between the diaphragm and the inferior vena cava is a longitudinal fiber cord, and the diaphragm is not a more easily identifiable tissue structure in imaging. This study confirmed that all tumor thrombi less than 3 cm below the level of the SPH could be removed transabdominally without combining cardiovascular surgeons and establishing CPB. In our group, there was one case with 34.51 mm tumor thrombus, and we transabdominally dissociated tissue to above the level of the tumor thrombus and completed the operation without establishing CPB. However, for a tumor thrombus near the atrial entrance, it is sometimes difficult to block and manage the inferior vena cava safely during the operation due to space constraints (Even if the IVC can be safely dissociated and suspended, the upper end of the IVC may slide down in the process of the head end descending of the tumor thrombus.), and patients with high Mayo IV tumor thrombus should prepare to establish CPB. The authors recommend that thrombectomy for high IVCTT should be routinely performed with sling suspension in the IVC below and above the SPH. When the height of the tumor thrombus is lower than the level of the sling above the SPH, we can occlude the subatrial IVC and stop CPB. When the height of the tumor thrombus is lower than the level of the sling below the FPH, we can release the FPH. The significant shortening of the operation time of CPB and FPH can greatly improve the safety of the operation and play a positive role in the postoperative recovery of blood coagulation, liver function and gastrointestinal function.

For a low Mayo III tumor thrombus with a height just reaching the level of the SPH, when patients are in the dorsal elevated position, there may be some natural drop in the tumor thrombus; strictly speaking, the preoperative Mayo grade of the tumor thrombus immediately changes from level III to II due to postural adjustment. In our group, there was 1 patient with 1 mm IVCTT above the SPH and 1 patient with 3.24 mm IVCTT above the SPH. The intraoperative ultrasound indicated that gravity caused the tumor thrombus to drop naturally to the inferior margin of the SPH; therefore, we did not dissociate and suspend the IVC above the SPH for two patients. The diameter of the tumor thrombus in thrombectomy for high tumor thrombus is also related to the difficulty of the operation [26, 27].

## Conclusions

In the preceding part of the paper, the author introduced in detail a new surgical strategy in which step-by-step and orderly lowering of the height of IVCTT is the key to thrombectomy. It is not hard to see that the current traditional Mayo grade cannot guide robotic operation for high IVCTT. In clinical practice, we found that the low supradiaphragmatic subatrial inferior vena cava can be safely transabdominally dissociated and suspended during the operation. This part of the level IV tumor thrombus can be removed simply by a transabdominal approach, and hepatobiliary surgeons and urologists can complete the operation in cooperation without the assistance of cardiovascular surgeons and the establishment of CPB. Therefore, the surgical management strategy is the same for level III tumor thrombus (above the SPH and subdiaphragm) and low level IV (tumor thrombus on the side of the diaphragm). They may be considered at the same level. The author agrees that the level of the SPH should be used as one of the anatomical markers in Mayo grade and recommends that the upper 3 cm level below the SPH would be more reasonable to replace the diaphragm as the boundary between level III and IV.

## Data Availability

The datasets used and/or analysed during the current study available from the corresponding author on reasonable request.
